# Cardiac Sarcoidosis: What’s Bad for the Ventricle is Bad for the Atria

**DOI:** 10.19102/icrm.2018.090205

**Published:** 2018-02-15

**Authors:** Rahul N. Doshi

**Affiliations:** ^1^Division of Cardiology, Keck School of Medicine, University of Southern California, Los Angeles, CA, USA

**Keywords:** Atrial fibrillation, cardiac sarcoidosis, ventricular tachycardia

In this month’s issue of *The Journal of Innovations in Cardiac Rhythm Management,* Lau et al. present an elegant case of cardiac sarcoidosis in a patient who presented first with atrial fibrillation and depressed left ventricular function and who subsequently underwent pulmonary venous isolation for the treatment of symptomatic atrial fibrillation.^1^ Based on a suspicious electrocardiogram and findings on cardiac imaging, the patient was diagnosed with cardiac sarcoidosis and underwent implantable cardioverter-defibrillator (ICD) placement for primary prevention. The patient then developed significant ventricular arrhythmias. Based on the presence of these arrhythmias and evidence of active inflammation on 2-deoxy-2-[fluorine-18]fluoro-D-glucose positron emission tomography scan, the patient was treated with immunosuppression therapy with resolution of the arrhythmia burden.

## Making the connection

I recall caring for a patient many years ago who first was referred to me with atrial fibrillation, conduction disease, and mild cardiomyopathy. Similar to the patient described in this current case report,^1^ my patient was treated with catheter ablation, pacemaker implantation, and then subsequent biventricular ICD placement after a biopsy-proven diagnosis was made. Within a few months, the patient developed both intractable heart failure and ventricular arrhythmias and, despite catheter ablation and aggressive hemodynamic support, eventually succumbed to multiorgan failure from cardiogenic shock.

My case at the time was quite sobering. Cardiac involvement in sarcoidosis can be fulminant and certainly points to active inflammation.^2^ Response to immunosuppressive therapy is certainly variable.^3^ It is well-established that, despite many years of experience with steroid use, there is no established benefit with regard to mortality.^2–4^ Lau et al. point out that early recognition and treatment is paramount to any potential favorable outcome.

## Considering other factors

It seems to be that, for classical cardiac disorders that result in arrhythmias, we often forget about the atria. Certainly, this may be based on the incidence of the type of arrhythmia as we understand it today. For example, in cardiac sarcoid, the classical sequelae are conduction system disease, ventricular arrhythmias, and heart failure.^4^ This association is based on the incidence of these findings when a diagnosis of cardiac sarcoidosis is made. The 2014 Heart Rhythm Society consensus statement does not include atrial arrhythmias in the diagnostic criteria for cardiac sarcoidosis. Yet, it is also well-established that asymptomatic cardiac involvement of sarcoidosis is highly prevalent.^5^ Could there be an overlap with a disease that is as highly prevalent as atrial fibrillation?

As the case by Lau et al. demonstrates, atrial fibrillation can be the first manifestation of cardiac sarcoidosis.^6^ Atrial fibrillation is the most common supraventricular arrhythmia associated with cardiac sarcoid.^7^ In patients with an established diagnosis of cardiac sarcoid, atrial fibrillation does occur less commonly than do ventricular arrhythmias^8^ but, again, many patients with cardiac sarcoidosis go undiagnosed.

There are many systemic or cardiac diseases that result in both atrial and ventricular arrhythmias. Other infiltrative disorders such as cardiac amyloid^9^ or arrhythmogenic right ventricular cardiomyopathy^10^ are associated with atrial fibrillation. There is good reason to be preoccupied with the risk of ventricular arrhythmias given the potential dire consequences. It is also easy to diagnose atrial fibrillation without considering the link to other potential diseases given the prevalence of the former. However, many of these biases also stem from our ability to detect involvement in the atria. Indeed, as cardiac imaging modalities continue to improve, we may be able to more reliably detect characteristic disease patterns in the atria as we commonly do in the ventricle.^11^

The coexistence of both ventricular and atrial arrhythmias are not unique to infiltrative disorders. The classical channelopathies, such as long QT syndrome or the Brugada syndrome, are associated with atrial fibrillation.^12^ The same genes responsible for these classically inherited disorders associated with ventricular arrhythmias are also implicated in familial atrial fibrillation.^13^ Observations such as these are important in our understanding of cellular electrophysiology; that is, the same structural or functional abnormalities that predispose the ventricular myocardium to arrhythmias can also affect the atria.

The case presented by Lau et al. illustrates several important lessons for the clinical electrophysiologist. First, disorders that create an arhythmogenic substrate in the ventricle may do so in the atria. It of course works both ways. A patient with cardiac sarcoidosis, which increases the risk of ventricular arrhythmias, may also have atrial arrhythmias. Conversely, a patient with cardiac sarcoidosis with atrial fibrillation may be at increased risk for ventricular arrhythmias. The presence of either kind of arrhythmia may be a marker of disease activity—in this case, noncaseating granulomas.

Secondly, with increasing resolution, we may be able to better delineate structural abnormalities associated with atrial fibrillation. Scar burden in the atria has been demonstrated as a factor that increases the likelihood of atrial fibrillation and to negatively affect ablation success.^14^ One could imaging that, with higher resolution, we might be able to evaluate distinctive patterns associated with particular disease states. One could even imagine that, given the variable penetrance associated with these diseases, we might someday be able to better characterize the true prevalence of these disorders and perhaps target treatment before clinical consequences develop.

Third, the assessment of disease activity (with both clinical events and diagnostic imaging) resulted in the present case undergoing effective treatment. Immunosuppressive therapy and/or perhaps antiarrhythmic drug treatment was effective at suppressing arrhythmia burden and, thus far, appears to slow down or halt disease progression.

In closing, one could argue that what’s bad for the ventricle, is bad for the atria. A disease that causes inflammation and scarring in the ventricle such as cardiac sarcoidosis can cause inflammation and scarring in the atria as well and result in atrial fibrillation. Disease activity in one may predict activity in the other. However, perhaps what’s good for the ventricle is also good for the atria. Treatment that halts the disease process in the ventricle may also suppress atrial fibrillation. It’s likely best that, when treating these disorders, we consider both possibilities.
